# 

**Published:** 1861-01

**Authors:** 


### CONTENTS FOR JANUARY, 1861.

### ORIGINAL COMMUNICATIONS.

Public Hygiene and Legal Medicine........ 1
Inversio-Uteri—its Causes—Mechanism and Medico-Legal Bearings........ 4
Diphtheria. By T. J. Pearce, M. D., of Decatur, Indiana........ 36
Clinical Lecture—Mercy Hospital. Service of Prof. N. S. Davis, M. D........ 38

### BOOK AND PAMPHLET NOTICES.

On Diseases Peculiar to Women. By Hugh L Hodge, M. D........ 46
An Elementary Treatise on Human Anatomy. By Joseph Leidy, M. D........ 46
Sixth Registration Report of South Carolina. By Robert W. Gibbs, Jr., M. D........ 46
The Pocket Anatomist. By H. W. Hilles........ 47
The Physician's Visiting List, Diary, and Book of Engagements, for 1861........ 47

### SELECTIONS.

Thoracentesis........ 47
Digitalis in Heart Disease........ 48
Cæsarean Section........ 49
Method of Shortening some Tedious Labors........ 49
Tincture of Benzoin for Chapped Nipple........ 50
Oxalate of Cerium in Vomiting........ 50
Out-Door Life for Phthisis........ 51
Mania a Potu........ 51
Treatment of Chorea........ 52
Treatment of Epilepsy........ 53

### PROCEEDINGS OF SOCIETIES.

Abstract of the Proceedings of tho Esculapian Society, at the Meeting held in Paris, Ill., January 1st and 2d, 1861........ 54

### EDITORIAL.

The American Medical Association........ 58
To the Members of the Illinois State Medical Society........ 60
Chicago Academy of Medical Sciences........ 63

